# Relationship of the levels of reactive oxygen species in the fertilization medium with the outcome of *in vitro* fertilization following brief incubation

**DOI:** 10.3389/fendo.2023.1133566

**Published:** 2023-03-06

**Authors:** Jianing Niu, Qiurong Chang, Junting Xu, Jinli Li, Wenqiang Liu, Zhiqin Chen, Xianting Jiao, Yi Guo, Xiaoming Teng

**Affiliations:** ^1^ Department of Assisted Reproduction, Shanghai First Maternity and Infant Hospital, School of Medicine, Tongji University, Shanghai, China; ^2^ Department of Obstetrics and Gynecology, Jiaxing Maternity and Child Health Care Hospital, College of Medicine, Jiaxing University, Jiaxing, China; ^3^ Department of Pediatric Cardiology, Xinhua Hospital Affiliated to Shanghai Jiao Tong University School of Medicine, Shanghai, China

**Keywords:** Fertilization medium, IVF outcome, MiOXSYS system, reactive oxygen species, sperm

## Abstract

Embryo selection in *in vitro* fertilization–embryo transfer (IVF-ET) mostly relies on morphological assessment using a conventional microscope or the time-lapse monitoring system, which is not comprehensive. Inappropriate levels of reactive oxygen species (ROS) in the fertilization medium may cause damage to gametes, eventually leading to adverse IVF outcomes. The present study aimed to identify the optimal oxidation–reduction level in the fertilization medium for IVF outcomes by measuring the static oxidation–reduction potential (sORP) using a highly accurate and sensitive MiOXSYS system. A total of 136 patients undergoing IVF following brief incubation were divided equally into 4 groups in this prospective cohort study. The sORP value in the fertilization medium was detected using the MiOXSYS system, and its relationship with IVF outcomes was analyzed. The primary outcome was pregnancy outcomes, including live birth rate (LBR), clinical pregnancy rate (CPR), biochemical pregnancy rate (BPR), and implantation rate (IR). The secondary outcome was embryo quality, including fertilization rate (FR), cleavage rate (CR), available embryo rate (AER), and good-quality embryo rate (GQER). Group II (sORP: 228.7–235.3 mV) showed a higher LBR, CPR, BPR, and IR compared with Group III (sORP: 235.4–242.7 mV), presented as follows: LBR (32.0% for Group II vs 3.6% for Group III, *P* = 0.033), CPR (32.0% for Group II vs 3.6% for Group III, *P* = 0.033), BPR (36.0% for Group II vs 3.6% for Group III, *P* = 0.019), and IR (31.3% for Group II vs 2.7% for Group III, *P* = 0.003). The FR in Groups I and II had lower significant differences compared with that in Groups III and IV (71.7% and 70.3% for Groups I and II vs 83.5% and 80.4% for Groups III and IV, *P* = 0.000). The GQER in Group I to Group IV was 32.7%, 37.4%, 26.5%, and 33.3%, respectively (*P* = 0.056). This study indicated that the sORP value in the fertilization medium might be a potential indicator of embryo quality and pregnancy outcome.

## 1 Introduction

Reactive oxygen species (ROS), including superoxide (O_2_^–^), hydroxyl radical (OH^−^), singlet oxygen (^1^O_2_), and hydrogen peroxide (H_2_O_2_), are produced by various biochemical cellular reactions, especially in the mitochondrial respiratory chain (1). Numerous mitochondria are retained during spermatogenesis to maintain various sperm functions while producing some ROS (2). A moderate level of ROS in spermatozoa regulates essential functional characteristics, such as motility, capacitation, and sperm–oocyte fusion (3). Oxidative stress occurs when the production of ROS exceeds the antioxidant scavenging ability, which has detrimental effects on sperm function, eventually affecting the fertility of an individual (4). In *in vitro* fertilization (IVF), the retrieved cumulus–oocyte complexes (COCs) are co-incubated with capacitated spermatozoa, which is a more physiological approach to selecting spermatozoa. Long-time spermatozoa–oocyte co-incubation results in an increase in the level of ROS in fertilization droplets because sperm may continue to produce ROS. A brief incubation of gametes is used to prevent the influence of excessive ROS on fertilization and embryo development. Some studies reported that a shorter co-incubation time was beneficial to improving embryo quality and clinical pregnancy (5, 6). However, other studies reported contradictory results (7, 8). Higher ROS levels in a blastocyst culture medium have been reported to lead to a lower blastocyst formation rate (BFR) (9).

The quality of embryos in IVF is mainly assessed by embryologists under an inverted microscope (10). Although clinical studies suggest that embryos with an appropriate number of blastomeres, good blastomere homogeneity, and less fragmentation may have a higher clinical pregnancy rate (CPR), identifying more objective indicators to optimize embryo selection is urgently required. One study reported that the ROS levels in day 3 embryo culture medium were negatively correlated with BFR and CPR (11). Another study reported that embryo quality, BFR, or CPR were not significantly influenced by the ROS levels in the culture medium (12). Considering that ROS detection in the fertilization medium does not affect day 3 fresh embryo transfer (ET), exploring the relationship between ROS levels in the fertilization medium and IVF outcomes is meaningful. Currently, the ROS levels in the medium are measured using the chemiluminescence assay, which is time-sensitive, time-consuming, and requires complex instrumentation and large sample volumes, complicating its application in routine diagnosis. The MiOXSYS system, which is based on a galvanic measure of electrons, has been used to measure the static oxidation–reduction potential (sORP) in human semen. ORP is described as an integrated measure of the balance between total oxidant activity (i.e., ROS, oxidized thiols, superoxide radicals, hydroxyl radicals, hydrogen peroxides, nitric oxides, peroxynitrites, transition metal ions, and so forth) and total reductant activity (i.e., free thiols, ascorbates, α-tocopherols, β-carotenes, uric acids, and so forth) (13). A high sORP value indicates an excess of oxidizing agent relative to the reducing agent, that is, an imbalance in the oxidation–reduction (REDOX) state. This method is time-saving, requires fewer samples, and can be monitored in real time (14, 15). This study aimed to investigate the correlation between REDOX level in the fertilization medium and IVF outcomes by detecting the sORP of the fertilization medium through a brief incubation of gametes.

## 2 Material and Methods

### 2.1 Study design, patients, and sample selection

This study was conducted between October 2019 and October 2020 at the Center of Reproductive Medicine of Shanghai First Maternity and Infant Hospital, Tong Ji University. A total of 136 patients attending the Center for brief IVF were screened and recruited. The inclusion criteria were as follows: female patients aged ≤35 years and participants undertaking *in vitro* fertilization–ET (IVF-ET) treatment for tubal factor, endometriosis, or mild male factor. The exclusion criteria were as follows: endometriosis stage III and IV; history of ovarian cystectomy; and polycystic ovary syndrome (PCOS). This study was approved by the ethics committee of Shanghai First Maternity and Infant Hospital, and written informed consent was obtained from each patient.

All participants included in this study underwent personalized controlled ovarian stimulation (COS). The fertilization medium was collected soon after the zygotes were removed, and sORP was subsequently evaluated using the MiOXSYS system.

### 2.2 Controlled ovarian stimulation

The patients underwent COS treatment by standard routines at the Center. The COS treatment included gonadotropin-releasing hormone (GnRH)-antagonist (GnRH-ant) protocol, GnRH-agonist (GnRH-a) protocol, and other protocols including mild stimulation protocol or progestin-primed ovarian stimulation (PPOS) protocol, as decided by clinicians. In the GnRH-ant protocol, GnRH-ant (cetrotide or ganirelix) was started at a dose of 0.25 mg on day 6 of stimulation or when at least one follicle reached a diameter of 12 mm until the trigger day. For the long GnRH-a protocol, GnRH-a (intramuscular triptorelin 1.25–1.88 mg single dose) was initiated in the mid-luteal phase of the prestimulation cycle for pituitary downregulation. Then, gonadotropin treatment was commenced on day 2 of the menstrual cycle. For the mild stimulation protocol or PPOS protocol, the participants received triptorelin (subcutaneous injection 0.1 mg/d), oral clomiphene citrate (50–100 mg q.d.), or oral dydrogesterone (10 mg b.i.d) on day 2 or 3 of the menstrual cycle, and gonadotropin was used at the same time. When at least one follicle with a diameter ≥18 mm was detected, recombinant human chorionic gonadotropin was administered as a trigger and then transvaginal ultrasound-guided oocyte retrieval was performed after 36 h.

### 2.3 IVF procedures

Semen specimens were analyzed according to the fifth edition of the World Health Organization guidelines, and sperm were collected by the swim-up method. The COCs were washed twice after 2–3 h of oocyte retrieval and then placed in 100 µL of G-IVF medium (Vitrolife, Sweden) to achieve a final concentration of 1–2 × 10^6^ spermatozoa/mL. Then, the fertilization dish containing oocytes and sperm was incubated in a 5% CO_2_ humidified incubator at 37°C for 4 h. After the co-incubation of oocytes and sperm for 4 h, the oocytes were removed and placed in new drops of the G-IVF medium. After the oocytes were removed, 30 μL of the fertilization medium was collected and used for the sORP detection by the MiOXSYS system.

### 2.4 Measurement of sORP

The sORP was measured using a galvanostat-based MiOXSYS system (Aytubio Science, USA). Briefly, 30 μL of the fertilization medium was added to the sample port of the MiOXSYS sensor. The sensor was then inserted into the MiOXSYS analyzer for 2 min for analysis. Next, the sORP values were automatically displayed on the analyzer screen in millivolts (mV).

### 2.5 Embryo evaluation

Normal fertilization was confirmed 18–20 h after insemination by the presence of two pronuclei and two polar bodies. The cleavage-stage embryos were graded as Grade 1 to Grade 6 according to the number and symmetry of blastomeres, as well as the proportion of cytoplasmic fragments (10). Grade 1 embryos implied symmetrical blastomeres and no cytoplasmic fragments; Grade 2 embryos denoted symmetrical blastomeres and less than 25% fragmentation; Grade 3 embryos indicated asymmetrical blastomeres and no cytoplasmic fragments; Grade 4 embryos implied asymmetrical blastomeres and less than 25% fragmentation; and Grades 5 and 6 embryos denoted asymmetrical blastomeres and more than 25% and 50% fragmentation, respectively. Grade 1 or 2 embryos with six to nine cells were regarded as good-quality embryos, and all frozen and transferred embryos were defined as available embryos.

### 2.6 ET, cryopreservation, and frozen-thawed ET

In the fresh ET cycle, fresh embryo was transferred under transabdominal ultrasound guidance on day 3 after fertilization. On the day of ET, surplus Grades 1 and 2 embryos were cryopreserved using the vitrification kit (Kato Corp., Shizuoka, Japan) following the manufacturer’s protocols. The patients who did not undergo a fresh ET cycle received frozen ET (FET) 2 months after the fresh ET cycle if they had at least one frozen embryo. Embryo thawing was also performed using the vitrification kit (Kato Corp.) following the manufacturer’s protocols. The patients who underwent fresh ET cycle or FET received either vaginal or intravenous luteal support. The patients who confirmed a biochemical pregnancy 2 weeks after transplantation received a transvaginal ultrasound scan after 4 weeks to identify the presence of a gestation sac with a fetal heart, signifying clinical pregnancy.

### 2.7 Definition of primary and secondary outcomes

The primary outcome was pregnancy outcomes, including live birth rate (LBR), clinical pregnancy rate CPR, biochemical pregnancy rate (BPR), and implantation rate (IR). The secondary outcome was embryo quality, including fertilization rate (FR), cleavage rate (CR), available embryo rate (AER), and good-quality embryo rate (GQER).

### 2.8 Statistical analysis

All continuous data were expressed as means ± standard deviation if the normal distribution was verified using the Kolmogorov–Smirnov test; otherwise, the median (interquartile range) was used for data description. The categorical variables were expressed as frequencies [percentage (%)]. The subgroup characteristics were compared using the analysis of variance or the Kruskal–Wallis test for continuous variables, and the chi-square test or Fisher’s exact test was used for comparing categorical variables.

The Spearman correlation analysis was conducted to investigate the linear correlation between sORP value and clinical outcomes. The embryo quality assessment and the clinical outcomes of total transfer cycles among the four sORP groups were compared using the chi-square test, and *post hoc* pairwise comparisons were conducted using the Bonferroni test. Statistical analysis was performed using SPSS software (v22; IL, USA). A two-sided *P* value <0.05 indicated a statistically significant difference.

## 3 Results

### 3.1 Demographic and clinical characteristics of patients

The Spearman correlation analysis was performed for the relationship between sORP and embryo quality as well as clinical outcomes, and no linear correlation was found (**Table S1**). A total of 136 patients were divided equally into 4 groups to explore an exact range of sORP value with a guiding or predicting value for IVF outcomes, arranged in sORP order as follows: Group I (*n* = 34, sORP: 191.5–228.4 mV), Group II (*n* = 34, sORP: 228.7–235.3 mV), Group III (*n* = 34, sORP: 235.4–242.7 mV), and Group IV (*n* = 34, sORP = 242.8–295.3 mV).

The demographic and clinical features of the patients are presented in **Table 1**. The demographic and clinical characteristics were homogeneous among the four groups, including age, body mass index, infertility duration, and stimulation protocol. The ovarian reserve in female patients included basic FSH, antral follicle count, and anti-Mullerian hormone. The semen quality assessment in male patients included progressive motility, forward movement sperm number, and DNA fragmentation index.

**Table 1:** Demographic and characteristics of patients.

Characteristic	Group I (N=34)		Group II (N=34)		Group III (N=34)		Group IV (N=34)		P-Value
	No.	Value	No.	Value	No.	Value	No.	Value	
Age (yr)									
Male		31.38 ± 0.48		32.68 ± 0.71		31.88 ± 0.56		31.91 ± 0.73	0.541
Female		30.97 ± 0.34		31.24 ± 0.34		31.03 ± 0.47		31.03 ± 0.55	0.975
**BMI**		21.53 ± 0.36		21.54 ± 0.55		21.58 ± 0.46		20.80 ± 0.41	0.576
Infertility status									
Duration (yr)		2.53 ± 0.34		2.69 ± 0.32		2.72 ± 0.29		2.47 ± 0.26	0.922
Primary (No.%)		20 (58.8)^d^		22 (64.7)^d^		16 (47.1)		9 (26.5)^a,b^	**0.009**
Secondary (No.%)		14 (41.2)		12 (35.3)		18 (52.9)		25 (73.5)	
Stimulation protocol (No.%)									
GnRH agonist		13 (38.2)		16 (47.1)		16 (47.1)		13 (38.2)	0.880
GnRH antagonist		9 (26.5)		11 (32.4)		7 (20.6)		13 (38.2)	
Mild-stimulation protocol		5 (14.7)		3 (8.8)		5 (14.7)		3 (8.8)	
PPOS		7 (20.6)		4 (11.8)		6 (17.6)		5 (14.7)	
Laboratory test									
Female:									
FSH level on 3rd day (mIU/ml)		6.97 ± 0.58		6.93 ± 0.60		7.02 ± 0.61		7.01 ± 0.40	0.999
AFC	21	13.00 ± 1.21	18	13.72 ± 1.21	25	13.12 ± 1.29	20	14.80 ± 1.12	0.723
AMH (ng/ml)	21	3.81 ± 0.41	29	3.38 ± 0.36	22	4.55 ± 0.97	18	3.74 ± 0.65	0.564
Male:									
PR (%)		41.66 ± 2.69		42.63 ± 2.41		43.68 ± 2.90		41.46 ± 2.23	0.925
FMSN		3154.96 ± 475.15		4302.58 ± 713.85		4284.48 ± 692.49		4878.43 ± 680.76	0.303
DFI (%)		8.02 ± 0.55		9.83 ± 1.08		8.98 ± 1.10		9.75 ± 1.07	0.515
Sex hormone level on hCG day									
LH (IU/ml)	31	2.30 ± 0.38	34	3.85 ± 1.39	33	2.92 ± 0.48	33	4.37 ± 2.05	0.680
Estradiol (pg/ml)	31	2826.73 ± 350.22	34	1893.14 ± 240.61^c^	33	4108.22 ± 658.70^b,d^	33	2444.89 ± 292.22^c^	**0.002**
Progesterone (ng/l)	31	1.88 ± 0.52	34	0.91 ± 0.10	33	1.10 ± 0.11	33	1.01 ± 0.11	**0.045**

Values are mean ± SD or No.(percentage)

BMI, Body mass index; FSH, follicle-stimulation hormone; AFC, Antral follicle count; AMH, Anti-mullerian hormone; PR, Forward motile sperm percentage; FMSN, Forward movement sperm number; DFI, DNA fragmentation index; LH, Luteinizing Hormone.

Post-hoc comparison was conducted using the Bonferroni test.

P-value considered significant was in bold.

^a^p<0.05 compared with group I; ^b^p<0.05 compared with group II; ^c^p<0.05 compared with group III; ^d^p<0.05 compared with group IV.

If the data was partly missing in the analysis, the available number of patients was presented.

### 3.2 Embryo quality assessment

The retrieved oocytes, FR, CR, AER, and GQER are shown in **Table 2**. The FR had lower significant differences in Groups I and II than in Groups III and IV (71.7% and 70.3% for Groups I and II vs 83.5% and 80.4% for Groups III and IV, *P* = 0.000). The GQER in Group I to Group IV was 32.7%, 37.4%, 26.5%, and 33.3%, respectively (*P* = 0.056) (**Table 2**).

**Table 2:** Embryo quality assessment among 4 groups.

	Group I	Group II	Group III	Group IV	Total	P-Value
Oocytes retrieved (No.)	357	263	425	397	1442	
Fertilization rate (No.%)	256 (71.7)^c,d^	185 (70.3)^c,d^	355 (83.5)^a,b^	319 (80.4)^a,b^	1115 (77.3)	**0.000**
Cleavage rate (No.%)	251 (98.0)	182 (98.4)	344 (96.9)	309 (96.9)	1086 (97.4)	0.639
Available embryo rate (No.%)	124 (49.4)	94 (51.6)	150 (43.6)	164 (53.1)	532 (49.0)	0.086
Good-quality embryos rate (No.%)	82 (32.7)	68 (37.4)	91 (26.5)	103 (33.3)	344 (31.7)	0.056

Values are No.(percentage).

Post-hoc comparison was conducted by Bonferroni test and Fisher’s exact probability was conducted while the sample size was less than 40 or theoretical frequencies were less than 5.

P-value considered significant was in bold.

^a^p<0.05 compared with group I; ^b^p<0.05 compared with group II; ^c^p<0.05 compared with group III; ^d^p<0.05 compared with group IV.

### 3.3 Assessment of clinical outcomes

A total of 105 patients underwent ET, and only the first transfer cycle was enrolled in **Table 3**. Group II delivered a higher LBR, CPR, BPR, and IR compared with Group III, presented as follows: LBR (32.0% for Group II vs 3.6% for Group III, *P* = 0.033), CPR (32.0% for Group II vs 3.6% for Group III, *P* = 0.033), BPR (36.0% for Group II vs 3.6% for Group III, *P* = 0.019), and IR (31.3% for Group II vs 2.7% for Group III, *P* = 0.003).

**Table 3:** Clinical outcomes of transfer cycles.

Characteristic	Group I	Group II	Group III	Group IV	P-Value
	(N=23)	(N=25)	(N=28)	(N=29)	
**One/two embryos (No./No.)**	16/7	18/7	19/9	23/6	0.796
**Biochemical pregnancy (No.%)**	3 (13.0)	9^b^ (36.0)	1^a^ (3.6)	5 (17.2)	**0.019**
Implantation outcome					
Implantation (No.%)	2 (6.7)	10^b^ (31.3)	1^a^ (2.7)	4 (11.8)	**0.003**
One/two embryos implanted (No./No.)	2/0	6/2	1/0	4/0	0.066
Pregnancy outcome					
Clinical pregnancy (No.%)	2 (8.7)	8^b^ (32.0)	1^a^ (3.6)	4 (13.8)	**0.033**
Singleton/Multiple Pregnancy (No./No.)	2/0	6/2	1/0	4/0	0.066
Delivery outcome					
Live birth (No.%)	2 (8.7)	8^b^ (32.0)	1^a^ (3.6)	4 (13.8)	**0.033**
Singleton/Multiple birth (No./No.)	2/0	7/1	1/0	4/0	0.059

Values are mean±SD, No.(percentage) or No./No.

In each sORP group, variables were analyzed in total transfer cycles by Chi-square test.

Post-hoc comparison was conducted by Bonferroni test and Fisher’s exact probability was conducted while the sample size was less than 40 or theoretical frequencies were less than 5.

P-value considered significant was in bold.

a p<0.05 compared with group II;

b p<0.05 compared with group III.

## 4 Discussion

This study evaluated the REDOX status in the fertilization medium using the MiOXSYS system to explore the relationship between ROS levels in the fertilization medium and IVF outcomes. The results revealed that the sORP of the fertilization medium (228.7–235.3 mV) was an optimal sORP range for IVF outcomes, which presented the highest LBR, CPR, BPR, and IR.

Most conventional assays for Oxidative Stress (OS)evaluation were designed only to detect a single oxidant and lacked a full-scale view of the REDOX status. sORP detection provided a snapshot of the current balance of overall oxidation or reduction capacity, which was convincing, rapid, and accurate. Therefore, we used the MiOXSYS system on the fertilization medium to get a reliable and convincing REDOX level. As expected, an optimal sORP range was located in Group II for achieving a better clinical outcome.

Nowadays, single-embryo ET is promoted for the safety of pregnant women, and an accurate and rapid method for selecting the best-quality embryo is urgently needed. The embryo scoring is based on the morphological system, which is not comprehensive. In recent years, researchers have been trying to determine the potential of embryonic development by detecting related molecular markers in the embryo culture medium, which is invasive and sensitive. Huo et al. showed that the difference in amino acids in the D3 embryo culture medium had a high accuracy in predicting the embryo implantation potential (16). Abreu et al. found that the D5 culture medium of morphologically inviable embryos had higher concentrations of interleukin 8 and tumor necrosis factor-alpha, which was associated with abnormal cell division and cell death (17). In addition, the ROS level in the D3 culture medium might serve as a biochemical marker for blastulation and clinical pregnancy (11). This study was the first to explore the relationship between ROS levels in the fertilization medium and IVF outcomes. If the ROS level in the fertilization medium can be one of the indicators to predict the clinical pregnancy outcomes, we can select embryos for transplantation by combining the ROS value of the fertilization medium and the morphological score of embryos. In this study, we found that Group II (sORP: 228.7–235.3 mV) had a higher GQER than other groups, but it was not statistically significant (*P* = 0.056). We speculated that it could be attributable to the small sample size of the study (*n* = 34 in each group). Group II had higher LBR, CPR, BPR, and IR values, suggesting that the ROS level in the fertilization medium should be maintained at an appropriate range, and extremely high or low levels may affect clinical outcomes. A similar study reported that ROS in media induced lipid peroxidation in sperm membranes, which enhanced spermatozoa binding to the zona pellucida (18). In addition, the FR in Groups I and II was significantly lower than that in Groups III and IV. Considering that the total number of samples was relatively small, we speculated that the fertilization rates in Groups I and II were significantly lower than those in Groups III and IV due to no-and low-fertilization in Groups I and II.

In the traditional IVF method, sperm and oocytes are incubated for 16–18 h. Long exposure of oocytes to spermatozoa can increase the damage caused by ROS, leading to short-term IVF (4–6 h co-incubation) (19). In this study, ROS was detected after 4 h. We next aimed to set more groups, including 4 h, 8 h, and 16 h, to observe the relationship between ROS levels at different time points and IVF outcomes.

This study had some limitations. First, short-term fertilization was not a routine treatment protocol in our Center, and was only used for patients with unexplained infertility. So, the sample size was still relatively small in this study. Second, sperm, oocytes, and granulosa cells were present in the fertilization medium, and the total number of sperm for one oocyte was imprecise (approximately 10,000–20,000). We failed to identify the relative contribution of ROS from these cells.

## Data availability statement

The raw data supporting the conclusions of this article will be made available by the authors, without undue reservation.

## Ethics Statement

The studies involving human participants were reviewed and approved by The Ethics Committee of Shanghai First Maternity and Infant Hospital. The patients/participants provided their written informed consent to participate in this study

## Author contributions

Y.G. and X.T. conceived and designed the study. Q.C. and J.L. contributed to the sample collection. J.X. analyzed the data. Q.C., Y.G. and J.N performed the experiments and wrote the manuscript. X.T., X.J. and W.L critically revised the manuscript. All authors provided a critical review and approved the final manuscript.
