# Supplementary material for: Relationship of the levels of reactive oxygen species in the fertilization medium with the outcome of in vitro fertilization following brief incubation
Source: Front Endocrinol (Lausanne). 2023 Mar 6;14:1133566. doi: 10.3389/fendo.2023.1133566 (PMC10025477; doi:10.3389/fendo.2023.1133566)
Supplement: Supplementary file 1 [file Table_1.pdf]

Table SI

The sORP value associated with embryo and clinical outcome

	Spearman correlation analysis	
	**Rs**	**P-Value**
Fertilization rate (%)	0.148	0.086
Cleavage rate (%)	-0.162	0.064
Available embryo rate (%)	0.092	0.294
Good-quality embryos rate (%)	0.049	0.575
Biochemical pregnancy	-0.108	0.275
Implantation	-0.080	0.418
Clinical pregnancy	-0.080	0.418
Live birth	-0.080	0.418
